# Down-regulation of long non-coding RNA ESCCAL_1 inhibits tumor growth of esophageal squamous cell carcinoma in a xenograft mouse model

**DOI:** 10.18632/oncotarget.23153

**Published:** 2017-12-11

**Authors:** Yuanbo Cui, Wei Wu, Pengju Lv, Jianying Zhang, Bingqing Bai, Wei Cao

**Affiliations:** ^1^ Translational Medicine Center, Zhengzhou Central Hospital Affiliated to Zhengzhou University, Zhengzhou, People’s Republic of China; ^2^ Helen Dillar Family Cancer Center, Department of Medicine, University of California in San Francisco, San Francisco, CA, USA; ^3^ Henan Academy of Medical and Pharmaceutical Sciences, Zhengzhou, People’s Republic of China; ^4^ Department of Clinical Medicine, Zhengzhou University, Zhengzhou, People’s Republic of China

**Keywords:** esophageal squamous cell carcinoma, long non-coding RNA, ESCCAL_1, tumor growth, phospho-kinase

## Abstract

Esophageal squamous cell carcinoma (ESCC) is one of the most lethal malignant cancers with high incidence and mortality. Current reliable effective diagnostic and prognostic biomarkers are very limited in clinic. Emerging evidence indicates that dysregulated expression of the long non-coding RNAs (lncRNAs) was examined in various types of cancer including ESCC. ESCC associated lncRNA _1 (ESCCAL_1) was first time identified to be increased expression in ESCC, and therefore named by our research team. However, its potential function in the progression of ESCC remains unclear. In this study, we investigated the effect of ESCCAL_1 knockdown on ESCC tumorigenicity using a xenograft mouse model and explored the underlying molecular mechanism. Here we showed that ESCCAL_1 knockdown significantly inhibited EC9706 cell growth in nude mice. Interestingly, we also found that reduced expression of ESCCAL_1 resulted in distinct alterations of relative phosphorylation level of kinases (p-p38α, p-JNK, p-FAK and p-Src), and significant changes of the expression level of apoptosis-related proteins (p53, BAX, Bcl-2 and Caspase-3). In summary, our results suggest that lncRNA ESCCAL_1 is a potential diagnostic and prognostic target of ESCC.

## INTRODUCTION

Esophageal squamous cell carcinoma (ESCC) is one of the most malignant cancers worldwide, and the predominant histologic type of esophageal carcinoma in Asia [1]. ESCC is the sixth leading cause of cancer-related death with more than 400,000 deaths each year [2–3]. There is obvious regional distinction in ESCC incidence, which mainly occurs in the “esophageal cancer belt” from northeast China such as Henan, Shanxi and Hebei provinces to the Middle East [2–3]. Despite the development of treatment for ESCC including chemotherapy, radiation therapy, surgery and preventive approaches, the majority of patients are diagnosed as ESCC at the advanced stage [4–5]. Five-year overall survival rate of ESCC remains less than 30% due to recurrence, drug resistance and metastasis [6–7]. Therefore, to explore novel effective biomarker and diagnostic and prognostic target for ESCC is an unmet need.

Long non-coding RNAs (lncRNAs) are new members of non-coding RNAs in the mammalian genome, they are over 200 nucleotides in length. In general, lncRNAs have no protein-coding capacity and lack a complete open reading frame (ORF) [8]. Although the transcripts of lncRNAs do not translate into proteins, lncRNAs play crucial roles in a variety of biological and physiological processes in regulating chromatin modification and gene expression profiling [9]. In recent years, lncRNAs have been reported to function as oncogenes or competing endogenous RNA (ceRNA) involving in proliferation, apoptosis, invasion and metastasis in many cancers [10–12]. To date, only a few functional lncRNAs in ESCC have been identified and the underlying mechanisms are not fully elucidated [13–15]. Esophageal squamous cell carcinoma associated long non-coding RNA transcript 1 (ESCCAL_1) was first identified in 2013 and named by our team after validation study [16]. We have previously demonstrated that expression of lncRNA ESCCAL_1 was significantly increased in ESCC and inhibition of ESCCAL_1 expression promotes apoptosis and decreasing invasion in ESCC cell lines [17]. However, the detailed molecular mechanisms of ESCCAL_1 on ESCC tumor growth *in vivo* remains poorly understood.

In present study, we evaluated the effect of ESCCAL_1 knockdown on ESCC tumorigenicity in a xenograft mouse model, and further investigated signaling pathways alterations in xenografted tumor samples using protein arrays.

## RESULTS

### Effect of ESCCLA_1 knockdown on ESCC tumorigenesis *in vivo*

**Figure 1:**
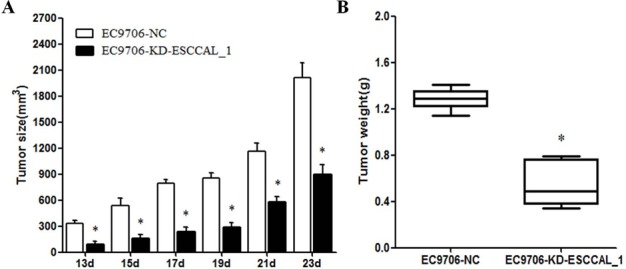
Effect of ESCCAL_1 knockdown on EC9706 cell growth in nude mice (**A**) Nude mice were subcutaneously injected with either mock siRNA expressed EC9706 cells (EC9706-NC) or ESCCAL_1 shRNA expressed EC9706 cells (EC9706-KD-ESCCAL-1). *N* = 6 for each group. Tumor volume was measured post-injection on 13 d, 15 d, 17d, 19d, 21d and 23d. (**B**) Tumor weight of xenografts tumors from sacrificed mice in two groups on day 23 was measured. Data are presented as mean ± SEM. *p < 0.05 is significant difference when compared KD-ESCCAL-1 with NC group of mice.

In order to investigate the effect of ESCCAL_1 knockdown on tumor growth, nude mice were injected subcutaneously with either EC9706 cells expressing ESCCAL_1 negative control (NC-EC9706) or EC9706 cells expressing ESCCAL_1-RNAi knockdown (ESCCAL_1-KD EC9706). Each group contains 6 mice. The tumor volume in KD group showed significantly reduced (*p <* 0.005) compared to the mice in NC group at six different time points [day 13 (*p =*0.001), day 15 (*p =* 0.004), day 17 (*p <* 0.001), day 19 (*p <* 0.001), day 21 (*p <* 0.001) and day 23 (*p <* 0.001) ] (Figure 1A). The tumor weight in KD group also displayed dramatically decreased compared to the mice in NC group on 23 d (*p <* 0.001) (Figure 1B). These results demonstrated that over-expression of ESCCAL-1 promotes tumor growth and down-regulation of ESCCAL_1 inhibits ESCC tumor growth *in vivo*.

### Identification of protein signaling pathways in ESCCAL_1-driven tumor growth

**Figure 2:**
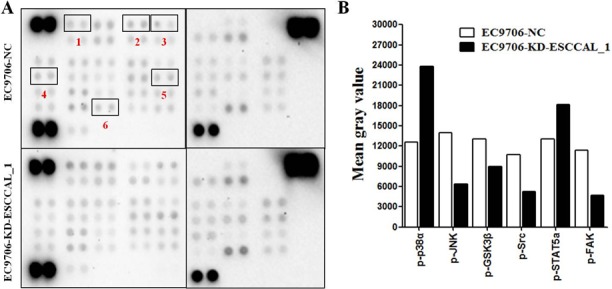
Screening of the phosphorylation levels of protein kinases in tumor tissues (**A**) Detection of phosphorylated proteins by using the phospho-kinase membrane array, the differential expression of six kinase proteins was highlighted with red box (1.p-p38α, 2.p-JNK, 3.p-GSK3β, 4.p-Src, 5.p-STAT5a, 6.p-FAK). (**B**) Quantification by mean gray value revealed the changes of relative phosphorylation levels of kinases in ESCCAL_1 KD group relative to NC group.

The process of tumorigenesis is complex and may involve in many signaling pathways associated with cell proliferation and survival. Given the complexity and possible cross-talk among these signaling pathways, a human phospho-kinase array was used to screen ESCCAL_1-mediated key signaling pathways during tumor formation in xenograft model. The total proteins from xenografted tumor samples of sacrificed mice (day 23) were subject to kinase protein phosphorylation detection on 45 protein array. The differential expression levels of kinase proteins between tumor samples from two groups were quantified for comparison. Our result showed that the relative phosphorylation levels of several kinases were increased or decreased between the two groups (Figure 2A). When compared with the NC group, the proteins levels of phosphorylated p38α (p-p38α) and phosphorylated signal transducer and activator of transcription 5a (p-STAT5a) in the KD group were elevated. We also observed that the proteins levels of phosphorylated c-Jun N-terminal kinase (p-JNK), phosphorylated focal adhesion kinase (p-FAK), phosphorylated glycogen synthase kinase 3β (p-GSK3β) and phosphorylated sarcoma (p-Src) were decreased in slowly growing tumor tissues following ESCCAL_1 knockdown (Figure 2A–2B). These data suggest that ESCCAL_1 knockdown predominantly decreased activities of protein kinases.

### ESCCLA_1 knockdown decreased phosphorylation of JNK1, FAK and Src

**Figure 3:**
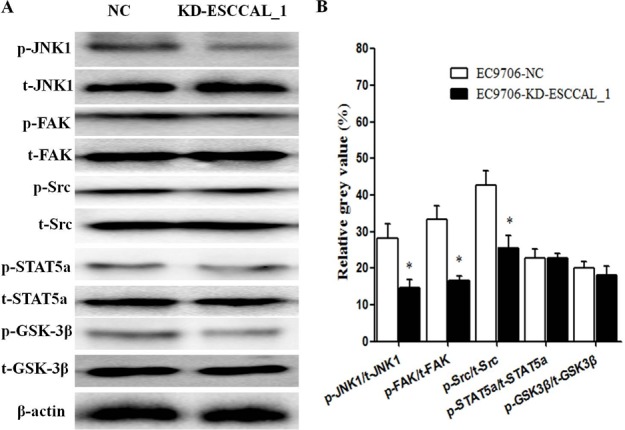
Down-regulation of ESCCAL_1 decreased protein levels of p-JNK1, p-FAK and p-Src confirmed by western blot assay (**A**) Total protein lysates from ESCCAL_1 KD group or NC group were subject to Western blot detection with different antibodies [p-JNK1, t-JNK1, p-FAK, t-FAK, p-Src, t-Src, p-STAT5a, total STAT5a (t-STAT5a), p-GSK3β and total GSK3β (t-GSK3β)]. (**B**) Quantitative protein levels of p-JNK1, p-FAK, p-Src, p-STAT5a and p-GSK3β were normalized with t-JNK1, t-FAK, t-Src, t-STAT5a and t-GSK3β, respectively. Data are presented as mean ± SEM. *n* = 5 per group. *p < 0.05.

We further verified this differential expression of kinase proteins in tumors derived from xenografts using conventional Western blot analysis. As shown in Figure 3, the proteins levels of total JNK1 (t-JNK1), total FAK (t-FAK) and total Src (t-Src) had not significant difference between ESCCAL_1-KD group and NC group. However, the expression levels of p-JNK1, p-FAK and p-Src were reduced significantly in the tumors after ESCCAL_1 knockdown when compared to the NC group (*p <* 0.001) (Figure 3A–3B). While Western blot results did not detect significant change of p-STAT5a and p-GSK3β expression in the tumor tissues from KD group when compared to NC group (*p* > 0.001) (Figure 3A–3B). Put them together, over-expression of ESCCAL_1 promotes tumor growth through activation of p-JNK1, p-FAK and p-Src-mediated signaling pathways.

### Over-expression of ESCCAL_1 activates survival pathway and decrease apoptosis

**Figure 4:**
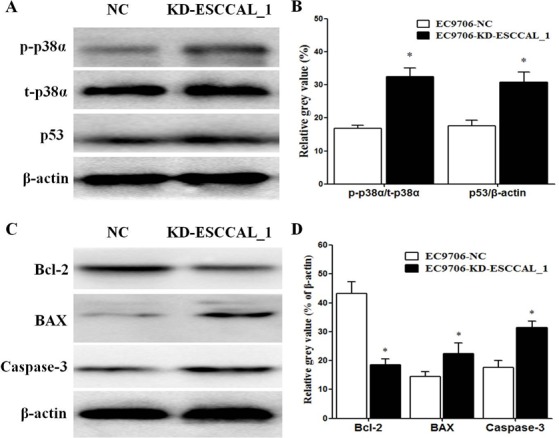
Over-expression of ESCCAL_1 promotes survival and suppresses apoptosis (**A**, **C**) Protein levels of p-p38α, total p38α (t-p38α), p53, Bcl-2, BAX and Caspase-3 were detected by Western blot. (**B**, **D**) Quantitative protein levels of p-p38α, p53, Bcl-2, BAX and Caspase-3 were normalized with t-p38α or β-actin. Data are presented as mean ± SEM. *n* = 5 per group. *p < 0.05.

In order to examine the effects of ESCCAL_1 knockdown on apoptotic signaling pathways, Western blot analysis was used to detect gene expressions invovling apoptosis and cell survival including p-p38α, p53, p21, Bcl-2, BAX and Caspase-3. Our data revealed that ESCCAL_1 knockdown significantly increased the phosphorylation of p38α, which was consistent with the result of phosphor-kinase array (*p <* 0.05) (Figure 4A–4B). We also noted that down-regulation of ESCCAL_1 could dramatically decrease the anti-apoptotic protein Bcl-2 and increase the pro-apoptotic proteins p53, BAX and Caspase-3 (*p <* 0.05) (Figure 4A–4D), but had no effect on p21 (data not shown). These data indicated that inhibition of ESCCAL_1 expression increased apoptosis and decreased survival in tumors.

## DISCUSSION

ESCCAL_1 is a novel lncRNA with oncogenic potential that was originally identified in ESCC using lncRNA array and bioinformatic analysis by our team [16]. Previous studies have shown that knockdown of ESCCAL_1 by small interfering RNAs could inhibit invasion and promote apoptosis in ESCC cell line EC9706 [17], high expression of ESCCAL_1 transcript was found to be correlated with poor prognosis of ESCC [18]. However, the specific role of ESCCAL_1 in ESCC tumorigenesis remains to be elucidated. In this study, we observed that knockdown of ESCCAL_1 could remarkably inhibit tumor growth of ESCC in a xenograft mouse model.

Consistent with our previous study that knockdown of ESCCAL_1 suppresses ESCC cell proliferation *in vitro* (data not shown), our result of tumor growth assay *in vivo* also revealed that down-regulation of ESCCAL_1 inhibits ESCC cell growth in xenografts nude mice. The phosphorylation status of kinase proteins directly reflects their activities, these activated proteins represent core cellular pathways including proliferation, differentiation, senescence and apoptosis [19]. So we screened multiple signaling pathways which might be affected by ESCCAL_1 knockdown in xenografts tumor tissues using phospho-kinase array. We found a series of phosphorylation of kinases proteins whose expression levels were either increased or decreased following ESCCAL_1 knockdown, these kinase proteins include p-p38α, p-JNK, p-GSK3β, p-Src, p-STAT5a and p-FAK. The phosphorylation levels of these kinases were verified independently by Western blot.

**Figure 5:**
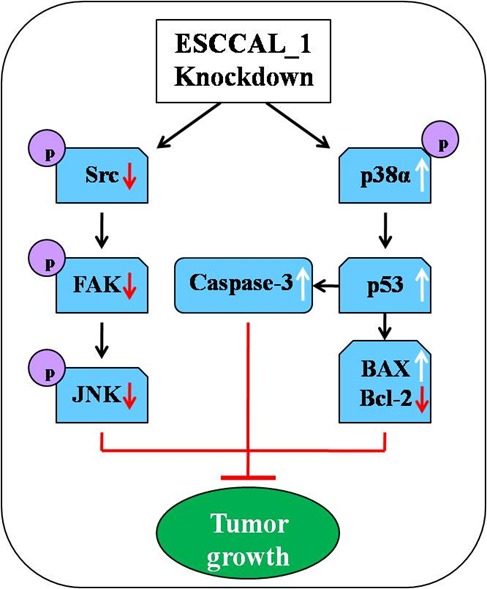
Diagram illustrates the underlying mechanism of ESCCAL_1 knockdown inhibits tumor growth in nude mice From the phenotype of overexpression of ESCCAL_1 promoting tumor growth to targets screening of phospho-kinase protein array, along with shRNA knockdown of ESCCAL_1 expression, the cross-talk of multiple signaling pathways may participate in ESCCAL_1-mediated oncogenesis. The inactivation of p-Src, p-FAK, p-JNK1, decrease of Bcl-2 expression and increase of p-p38, p53, BAX and Caspase-3 expression in ESCCAL_1 KD group were detected in this study (Red arrows: decrease; white arrows: increase; black arrows: activate/induce).

Previous study reported that up-regulation of GSK-3β contributed to an aggressive phenotype and cell proliferation in ESCC [20]. The STAT5a is the major isoform of STAT5 except STAT5b. It has been shown that both of the STAT5 isoforms were involved in many human cancers, and activation of STAT5a was closely related to tumor progression [21]. However, our data of Western blot showed that the phosphorylation of GSK-3β and STAT5a had no significant changes after ESCCAL_1 knockdown, suggesting that down-regulation of ESCCAL_1 inhibiting ESCC tumor growth involved in other signaling pathway. It has been reported that Src was aberrantly activated in many cancers and correlated to poor prognosis of non-small cell lung cancer and ESCC [22–23]. Increasing studies indicated that Src plays a key role in promoting tumor growth, invasion and metastasis via regulation of many downstream signaling molecules including FAK and JNK, which are widely considered to be crucial for cell proliferation and migration [23–24]. Our results displayed that the phosphorylation levels of Src, FAK and JNK were significantly decreased following ESCCAL_1 knockdown (Figure 5), suggesting that down-regulation of ESCCAL_1 inhibits tumor growth probably by inactivation of Src/FAK/JNK pathway.

The p38α pathway is known as a negative regulator in cell proliferation and tumorigenesis. Activation of p38α can inhibit tumorigenesis and tumor cell growth, which suggests that p38α is a tumor suppressor [25–26]. Tumor suppressor p53 plays an important role in regulating cell cycle and apoptosis in many cancers via repair of damaged DNA. If DNA repair fails, accumulation of p53 can stimulate the process of apoptosis to clear away the damaged cells. Previous study reported that activation of p38α induced increase of p53, thus triggered apoptosis and cell cycle arrest [27]. Consistent with this study, we found that down-regulation of ESCCAL_1 resulted in a significant increase of p-p38α and p53. Our data also showed that apoptosis associated proteins Caspase-3 and BAX were dramatically increased and Bcl-2 significantly decreased after ESCCAL_1 knockdown. Caspase-3 is a major executioner protein of apoptosis which can be activated by both extrinsic and intrinsic pathways. It has been shown that the p53 promotes apoptosis through interactions with Caspase-3 and Bcl-2 family proteins including BAX both *in vitro* and *in vivo* in recent studies [28–30]. According to our results, it can be speculated that down-regulation of ESCCAL_1 inhibits ESCC tumor growth might be mediated by Src and p38α pathway.

In summary, our data indicate that knockdown of ESCCAL_1 inhibits tumor growth in an ESCC xenografts mouse model, inactivation of Src and activation of p38α may be two cross-talk pathways responsible to the resulting phynotype. Our study also suggests that ESCCAL_1 is a putative oncogenic lncRNA during tumorigenesis.

## MATERIALS AND METHODS

### *In vivo* tumor growth assay

Six week old male BALB/c nude mice were purchased from the Shanghai Experimental Animal Center, Chinese Academy of Sciences (Shanghai, China). All experimental animal procedures were carried out according to the Ethical Committee of Zhengzhou University. All mice were housed under a 12 h light/dark cycle and automatically given food and water. In this experiment, the vector hU6-MCS-CMV-EGFP (GV155) (Genechem, China) was used to generate ESCCAL_1-shRNA or nonsense-shRNA (under human U6 promoter) according to the manufacture’s instruction, the shuttle vector and viral packaging system were cotransfected into HEK293T cells to replicate competent lentivirus [17]. The EC9706 cells infected with ESCCAL_1-shRNA or nonsense-shRNA Lentivirus were used for xenografts as our previously described [17]. Then, the nude mice were divided into two groups: Negative control (NC) (*n =* 6), injection of nonsense-shRNA Lentivirus infected EC9706 cells subcutaneously into the back flank; Knockdown (KD) (*n =* 6), injection of ESCCAL_1-shRNA Lentivirus infected EC9706 cells subcutaneously into the back flank. The tumor volumes were calculated as length × width^2^ × 0.5 from day 13 to day 23 every two days, the mice were sacrificed at day 23 after injection and tumor weights were measured for further studies.

### Tissue preparation

All the mice were sacrificed at day 23 after injection and the tumor tissues were immediately harvested. After rinsed in 0.9 % saline solution, the tissues were ground in the liquid nitrogen into a powder separately. Then, the total proteins were extracted from the tumor tissues by using RIPA lysis buffer containing PMSF (RIPA: PMSF = 100:1). Protein concentration of each tissue lysate was quantified by using a NanoDrop 2000 spectrophotometer (Thermo Scientific, USA). The proteins were stored in the refrigerator at –80°C or used for molecular biological analysis directly.

### Phospho-kinase antibody array

The Human Phospho-Kinase Array kit (R&D Systems, USA) was used to examine the expression of phosphorylated proteins in the tumor tissues according to the manufacturer’s instructions. This array specifically determines the relative phosphorylation levels of kinases and proteins associated with cell proliferation and apoptosis. Briefly, after blocking in the array buffer for 1 h at room temperature, the membranes were incubated with diluted proteins in each well overnight at 4°C on a rocking platform shaker. Then, the diluted detection antibody cocktail A or B was added into wells separately for part A or B membranes and incubated for 2 h at room temperature. After incubating with diluted streptavidin-HRP and chemi reagent mix for appropriate time, the immunoreactive dots on the membranes were detected by using the Chemidoc EQ system (BioRad, USA).

### Western blot analysis

Total proteins were denatured at 100°C by using a Thermocell cooling & heating block (Hangzhou Bioer Technology, China). Equal amounts of denatured proteins (100 μg) were separated by SDS-PAGE and transferred to a PVDF membrane. After blocking with 5% (weight: volume) skim milk for 1 h at room temperature on a rocking platform shaker, the membrane was incubated in the diluted primary antibody overnight at 4°C. Then, the membrane was incubated with HRP-conjugated secondary antibody for 2 h at room temperature. Finally, the immunoreactive bands on the membrane were detected by using the Chemidoc EQ system (BioRad, USA). Primary antibodies as follows: anti-STAT5a (1:200, Santa Cruz, USA), anti-p-STAT5a (1:200, Santa Cruz, USA), anti-FAK (1:200, Santa Cruz, USA), anti-p-FAK (1:200, Santa Cruz, USA), anti-GSK-3β (1:200, Boster, China), anti-p-GSK-3β (1:200, Santa Cruz, USA), anti-p38α (1:200, Santa Cruz, USA), anti-p-p38α (1:200, Santa Cruz, USA), anti-JNK1 (1:200, Santa Cruz, USA), anti-p-JNK1 (1:200, Boster, China), anti-Src (1:200, Boster, China), anti-p-Src (1:200, Santa Cruz, USA), anti-Caspase-3 (1:200, Santa Cruz, USA), anti-BAX (1:200, Boster, China), anti-Bcl-2 (1:200, Boster, China), anti-p53 (1:200, Boster, China), anti-β-actin (1:2000, Santa Cruz, USA).

### Statistical analysis

The data were analyzed by using the Graphpad Prism 5 software. All results are presented as Mean ± Standard error of measurement (SEM). Pairwise comparisons were carried out by using two-tailed student’s *t*-test. A value of *P <* 0.05 was considered to be statistically significant.
